# Effects of Surfactants on the Properties of Mortar Containing Styrene/Methacrylate Superplasticizer

**DOI:** 10.1155/2014/942978

**Published:** 2014-05-19

**Authors:** El-Sayed Negim, Latipa Kozhamzharova, Jamal Khatib, Lyazzat Bekbayeva, Craig Williams

**Affiliations:** ^1^Faculty of Science and Engineering, University of Wolverhampton, Wulfruna Street, Wolverhampton WV1 1LY, UK; ^2^Polymers and Pigments Department, National Research Centre, Dokki, Giza 12622, Egypt; ^3^Taraz State University Named After M.H. Dulati, 60 Tole Bi Street, 080000 Taraz, Kazakhstan; ^4^Distance of Education, Universiti Sains Malaysia, 11800 Penang, Malaysia

## Abstract

The physical and mechanical properties of mortar containing synthetic cosurfactants as air entraining agent are investigated. The cosurfactants consist of a combination of 2% dodecyl benzene sodium sulfonate (DBSS) and either 1.5% polyvinyl alcohol (PVA) or 1.5% polyoxyethylene glycol monomethyl ether (POE). Also these cosurfactants were used to prepare copolymers latex: styrene/butyl methacrylate (St/BuMA), styrene/methyl methacrylate (St/MMA), and styrene/glycidyl methacrylate (St/GMA), in order to study their effects on the properties of mortar. The properties of mortar examined included flow table, W/C ratio, setting time, water absorption, compressive strength, and combined water. The results indicate that the latex causes improvement in mortar properties compared with cosurfactants. Also polymer latex containing DBSS/POE is more effective than that containing DBSS/PVA.

## 1. Introduction

Polymer-modified mortars and concretes such as polymer latexes, redispersible polymer powders, water-soluble resins, and epoxy resins have been widely used in the world [1]. Since then, polymers have been added to mortars and concretes to enhance some properties including workability, water retention, permeability, strength, and dimensional stability [2]. Polymer modified mortars using latex are widely used as high performance, low-cost construction materials particularly for finishing work because of their enhanced workability, mechanical and durability properties [3–6]. Polymer latex is a colloidal dispersion of small polymer particles in water, which is generally produced by the emulsion polymerization of monomers with surfactants. Surfactants are a large group of surface-active substances with numerous applications because of their relatively complex behaviors [7–10]. Surfactants have a hydrophobic part and a hydrophilic part. Depending on the nature of the hydrophilic part the surfactants are classified as anionic, nonionic, cationic, and zwitterionic [11].

Ouyang et al. [12] studied the effect of the synthetic surfactant combining nonionic and anionic surfactant on the compressive strength of cement mortar. The results indicated that the suitable dosage of this surfactant could improve not only the fluidity but also the compressive strength of mortar. On the other hand, an excess amount of surfactant may have an adverse effect on the strength of the latex modified mortar and concrete because of the reduced latex film strength [2].

Boutti et al. [13] studied the influence of low dosage of styrene/butyl acrylate (St/BuA) latexes on some properties of cement mortars. The results showed that the use of anionic surfactant in the preparation of latex is highly detrimental to the workability. However, using latex with nonionic surfactant improved the workability due to the steric repulsion forces.

Styrene/methacrylate copolymers play a key role in the family of polymers latex used in Portland cement [14–16]. Negim et al. [17] showed that the rheological, physical, and mechanical properties of pastes are improved by using styrene/methacrylate polymer in the presence of different cosurfactants. These included DBSS/PVA and DBSS/POE.

In the present work, synthetic cosurfactants combining DBSS and either PVA or POE were selected as an air entraining agent, and the effect of copolymers latexes St/BuMA, St/MMA, and St/GMA on the physicomechanical properties of the cement mortar was investigated.

## 2. Experimental

### 2.1. Materials

Dodecyl benzene sodium sulfonate (DBSS) was used as anionic surfactant with a molecular weight of 348.48 g/mole. The nonionic surfactants used were polyvinyl alcohol (PVA) and polyoxyethylene monomethyl ether (POE) with a molecular weight of 13,000 and 5000 g/mole, respectively. The chemical structure of the various surfactants is shown in Table 1.

The raw materials used in the present study are Portland cement clinker (PCC) and raw gypsum (G). Each of those raw materials was separately ground in a steel ball mill until the surface area of, respectively, 3650 and 2800 cm^2^/g was achieved. The chemical composition of the raw materials is shown in Table 2. The mineralogical composition of the PCC sample is C_3_S, 58.79%; *β*-C_2_S, 17.68%; C_3_A, 8.08%; C_4_AF, 9.72%. The Portland cement (PC) was prepared by mixing 96% PCC and 4% G (by weight) in a porcelain ball mill for one hour using 3 balls to ensure complete homogeneity of the cement. The Blaine surface area of the cement sample was 3350 cm^2^/g [18].

The fine aggregate used was sand with particle size ranging from 0.21 mm to 0.53 mm and is free from organic or clay-like materials.

### 2.2. Synthesis and Characterization of Copolymers

Copolymer emulsion latexes based on styrene (St) with each of butyl acrylate (St/BuA), butyl methacrylate (St/BuMA), methyl methacrylate (St/MMA), and glycidyl methacrylate (St/GMA) were synthesized with composition ratios (5 : 5) using potassium persulfate/sodium metabisulfite (KPS/NaMBS) as redox initiation system in the presence of a coemulsifier that consists of 2% dodecyl benzene sodium sulfonate with 1.5% polyvinyl alcohol (DBSS/PVA) and 2% DBBS with 1.5% polyoxyethylene glycol monomethyl ether (DBBS/POE). The preparation of copolymers and the methods of analysis (^1^H NMR, rheological and morphological techniques) have been previously described in a previous investigation [19].

### 2.3. Mixing and Testing

Mortar specimens of size 70 mm cube were prepared in three groups. The control mix (M0) consists of Portland cement (PC), sand, and water. The proportion of cement to sand was 1 : 3 (by weight) and the water/cement ratio (w/c) was 0.45. In mixes M1 and M2, synthetic surfactants DBSS/PVA and DBSS/POE were added, respectively, whereas in mixes M3 to M8, prepared latexes were added. The addition rate was 2% by weight of cement. The cement to sand ratio was kept constant. However the water to cement ratio was changed so that the same consistency was achieved. Further details about the mixes are given in Table 3.

The cement and sand were intermixed until homogeneity was achieved. Then the surfactants or prepared latexes were added to the mixing water. This was then added gradually to cement/sand mixture in order to determine the water of consistency and setting time using Vicat apparatus [20, 21].

The resulting mortar was directly placed into 70 mm cube stainless steel moulds. The moulds were manually agitated for 2 minutes and then on a vibrator for another 2 minutes. The moulds were kept in a humidity chamber at 100% R. H and a constant room temperature overnight and then demoulded and cured under water till the time of testing. Testing included compressive strength, water absorption, and combined water and was conducted at 1 day and 3, 7, and 28 days. The determination of water absorption as per the specifications of BS 1881: Part 122 [22], compressive strength, water absorption, and combined water were described in a previous investigation by the authors [23].

## 3. Results and Discussion

### 3.1. Structure of Copolymers

**Scheme 1:**
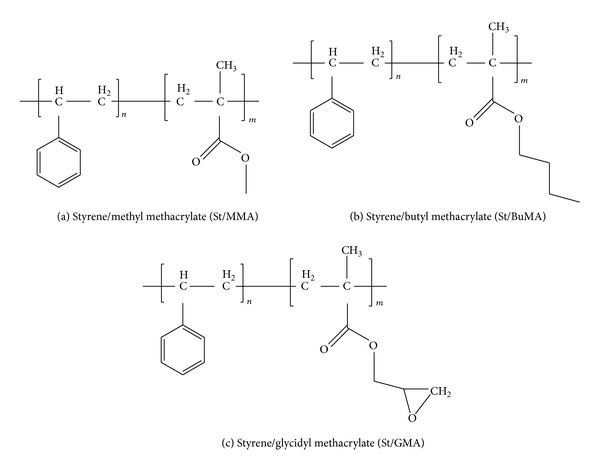
The chemical structure of copolymer latexes.

The structure of the copolymers (St/BuMA, St/MMA and St/GMA) is shown in Scheme 1 and further details about the synthesis and characterization have been previously reported by the authors [19].

### 3.2. Flow Table

**Figure 1:**
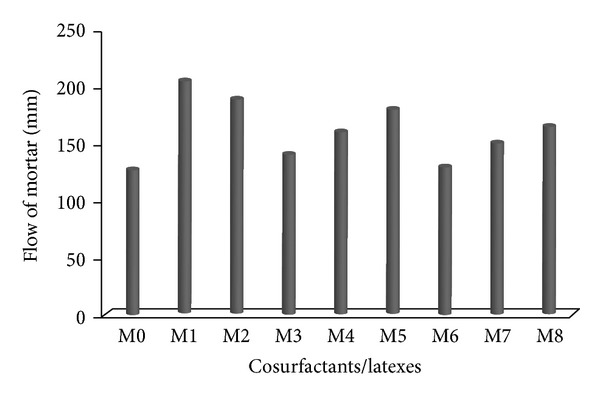
The effect of cosurfactants and copolymer latexes on the flow of mortar.

**Figure 2:**
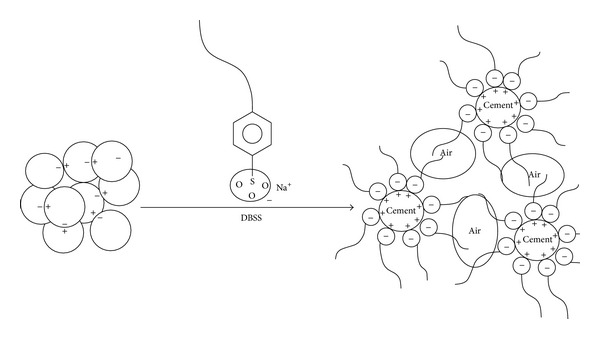
Mechanism of anionic surfactant (DBSS) with negative charge in mortar acting as an air entrainment.

The effect of cosurfactants and copolymer latexes on the flow of Portland cement mortar is shown in Figure 1. It can be seen that the addition of cosurfactants to mortar increased the flow. For the reference mortar (M0) the flow was 128 mm whereas mortar containing surfactant DBSS/PVA (M1) and surfactant DBSS/POE (M2) had a flow of 205 mm and 190 mm, respectively. It was observed that mortar with surfactant DBSS/PVA and DBSS/POE had large volume of air bubbles that may have contributed to the increase in the flow. These quantities of air bubbles are the results of anionic action of surfactants that have a hydrophobic and a hydrophilic end as shown in Figure 2. The hydrophobic end reduces the surface tension and allows the formation of stable bubbles. Also the hydrophilic end is attracted by charges on the surface of cement and sand particles. This attaches the bubble to the surface and helps to produce a stable structure in the mix (Figure 2). On the other hand, the flow of mortar premixed with latexes in presence of cosurfactants DBSS/PVA (i.e., M3–M5) is higher than that of latexes in presence of cosurfactants DBSS/POE (M6–M8) and the reference mortar (M0), respectively, as shown in Figure 1. This is mainly interpreted in terms of improved consistency or fluidity due to the ball bearing action of polymer particles and the dispersing effect of surfactants in the latexes among cement particles [24, 25]. The dispersing effect of surfactants is generally dependent on the monomer composition, the type, and concentration of surfactants [26, 27]. Generally, latex-modified mortar provides a good workability over conventional cement mortar. The workability is further increased in the presence of PVA [28–30].

### 3.3. Water of Consistency and Setting Time

**Figure 3:**
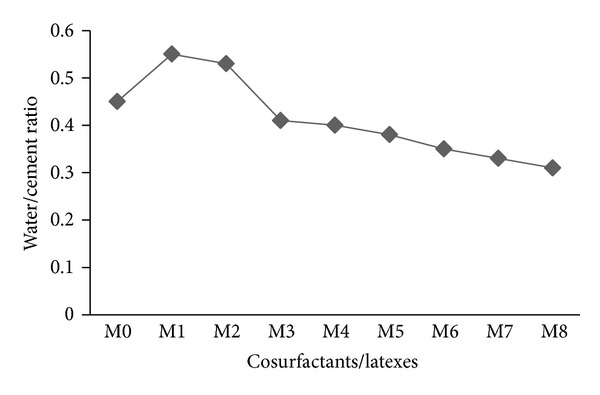
The effect of cosurfactants and copolymer latexes on the water/cement ratio of mortar.

**Figure 4:**
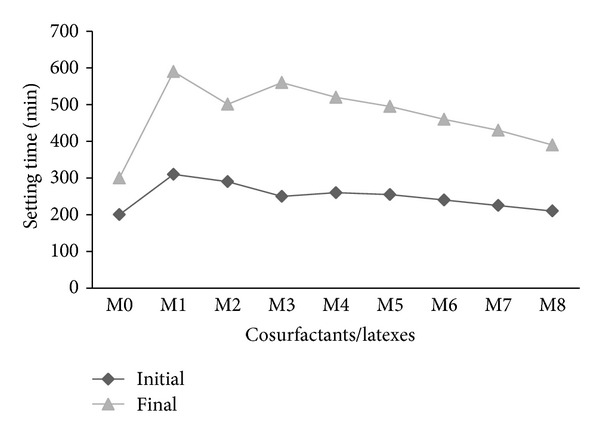
The effect of cosurfactants and copolymer latexes on the setting time of mortar.

Figures 3 and 4 show the water/cement ratio required to achieve the same consistency and the setting time, respectively, for all mortar mixes with synthetic cosurfactants and copolymer latexes prepared with two different cosurfactants. As it is well documented that the water/cement ratio is directly related to the mechanical and physical properties of cement mortar. Compared with the reference mortar (M0), the water/cement ratio increases with the addition of cosurfactants (M1 and M2) and decreases with the presence of latexes (M3–M8). However, the water/cement ratio of mortar decreases with the addition of latexes in presence of DBSS/PVA in the following order M5 < M4 < M3 and in case of DBSS/POE in the following order M8 < M7 < M6 as shown in Figure 3. Furthermore, the reduction of water/cement ratio using copolymers in the presence of POE was more than that in case of PVA. This is attributed to some chemical reactions that may take place between the particle surfaces of reactive copolymers and silicate surfaces over the aggregates [1]. Such reactions are expected to reduce the water/cement ratio [31]. A reduction in water requirement was expected not only due to the presence of surfactants in the polymers but also due to the lower surface tension of polymer molecules, which facilitates better flow of the mix at the same water content.

The initial and final setting times of mortar mixes with cosurfactants (M1 and M2) were longer than those mixed containing copolymer latexes (M3–M8) prepared in presence of cosurfactants as shown in Figure 4. This may be attributed to the presence of the surfactants such as alkylbenzene sulfonates and polyvinyl alcohol contained in latexes, which inhibit the hydration of mortar as reported elsewhere [1]. However, the increase in setting times of mortar premixed with latexes in presence of DBSS/PVA was higher than those latexes containing DBSS/POE. A previous investigation by the authors on cement pastes showed an opposite trend [17]; pastes containing latexes in the presence of cosurfactant DBSS/PVA had shorter setting times than those containing DBSS/POE [17].

Apparently from Figure 4, mortar containing St/MMA with shorter side chain length shows longer setting time than that of St/BuMA and St/GMA, respectively. The setting time of mortar may be varied according to the physical and chemical properties of organic polymers such as solubility, viscosity, chain length, polarity, and functional groups [32].

### 3.4. Water Absorption

**Figure 5:**
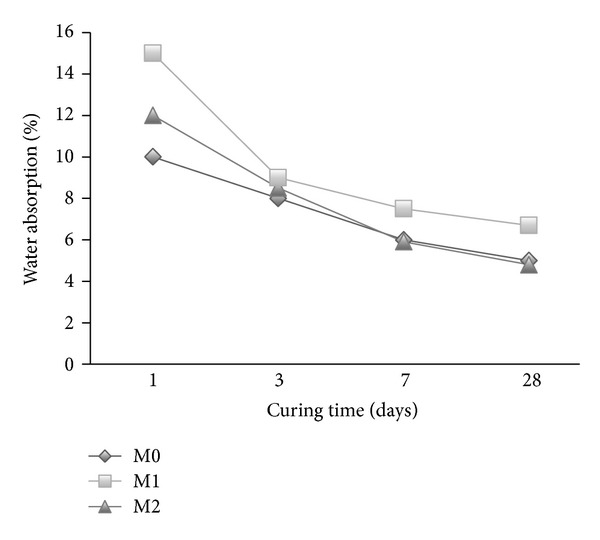
The effect of cosurfactants on the water absorption of mortar.

**Figure 6:**
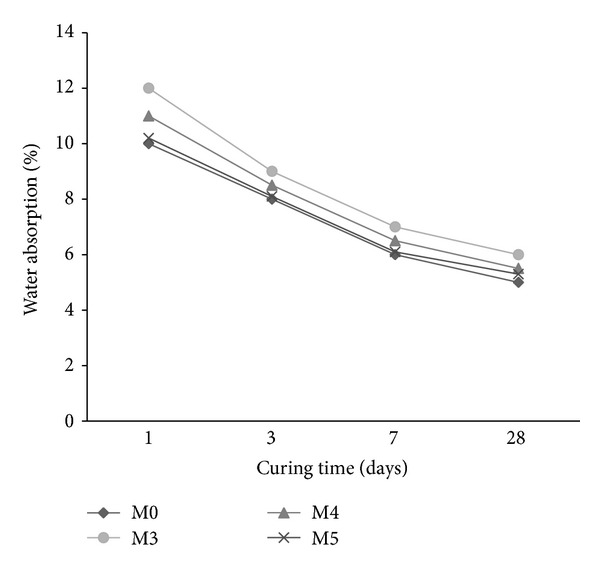
The effect of copolymer latexes prepared in presence of DBSS/PVA on the water absorption of mortar.

**Figure 7:**
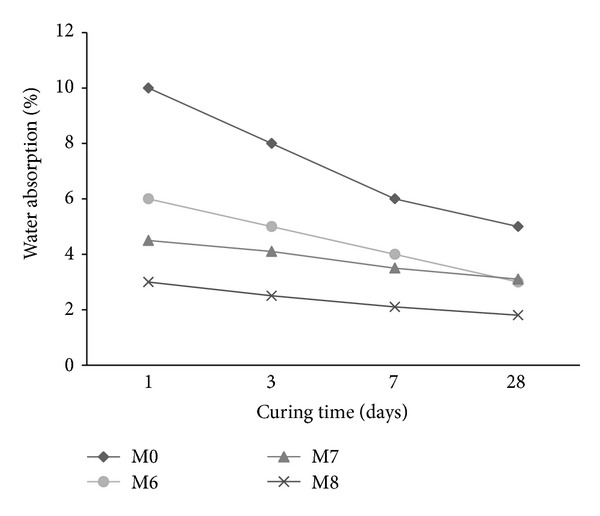
The effect of copolymer latexes prepared in presence of DBSS/POE on the water absorption of mortar.

The water absorption of mortar mixes is shown in Figures 5–7 in the presence of cosurfactant, copolymer latexes with PVA and POE, respectively, at different curing times. The absorption reduces with the increase in curing time for all mixes. There is an increase in water absorption in the presence of cosurfactants (M1 and M2) and copolymer latexes containing PVA (M3–M5) (Figures 5 and 6). By contrast, the water absorption of mortar decreases with the addition of copolymer latexes in the presence of POE (Figure 7). The increase of water absorption may be due to the increase in bubbles content, which in turn increases the porosity. However, mortar mixes with copolymer latexes containing POE (M6–M8) have lower water absorption than those containing PVA (M3–M5) and the reference mortar mix (M0). The decrease in water absorption may be due to the formation of polymer film that may have sealed the pores and inhibited the penetration of water [33–35]. The water absorption of mortars premixed with St/GMA is less than that of St/BuMA and St/MMA, respectively. This is due to the presence of epoxy group in the GMA, which has greater effect on reducing water penetration, compared to mortars mixed with BuMA and MMA.

### 3.5. Compressive Strength

**Scheme 2:**
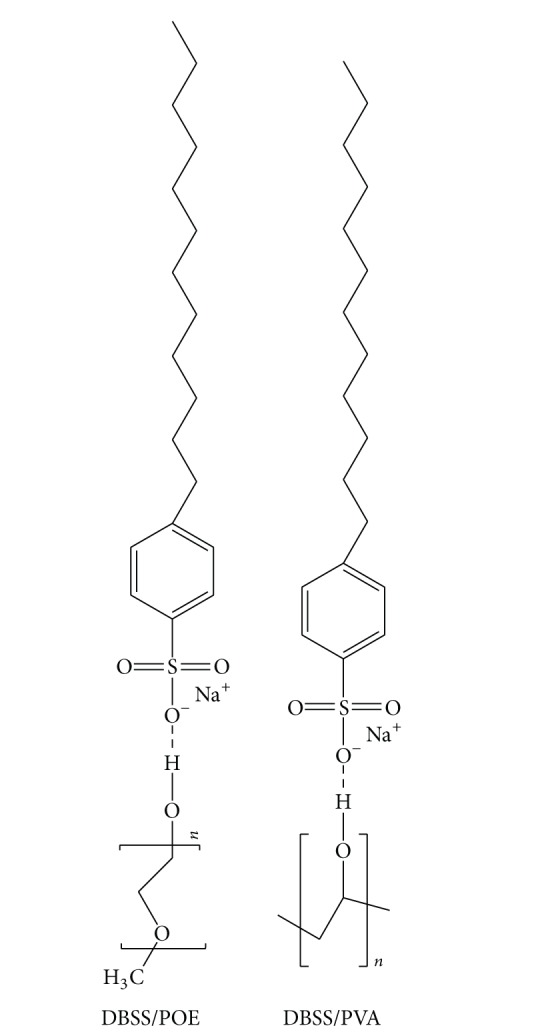
Formation of hydrogen bond between DBSS and POE and PVA.

**Figure 8:**
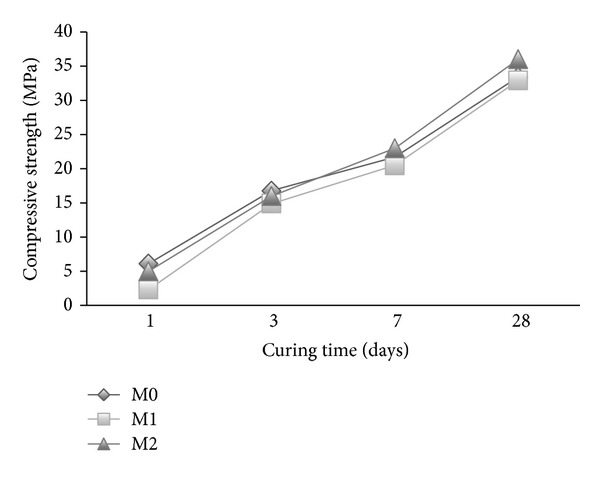
The effect of cosurfactants on the compressive strength of mortar.

The compressive strength of mortar containing cosurfactants (M1 and M2) is shown in Figure 8 for different curing times. The use of cosurfactants resulted in little variation in strength compared with the reference mortar mix (M0). This indicates that using cosurfactants in mortar improves the compressive strength compared with mortar containing one surfactant only [1, 29]. It is found that when DBSS interacts with either PVA or POE, there is a new hydrogen bond (Scheme 2) forming that fills the pores which improves the bond between the paste and aggregate, thus resulting in strength enhancement [30, 36].

**Figure 9:**
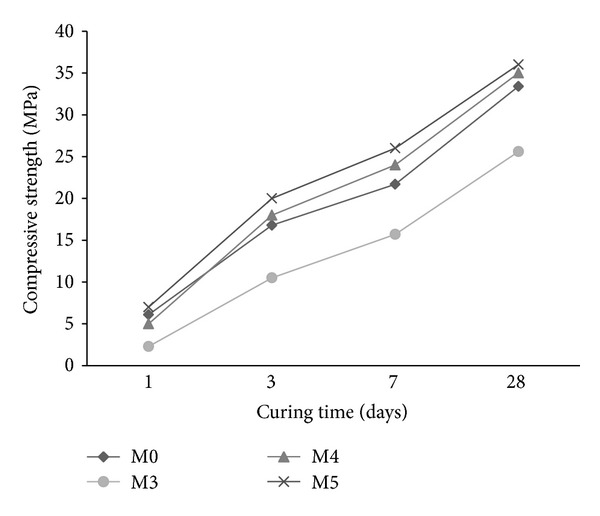
The effect of copolymer latexes prepared in presence of DBSS/PVA on the compressive strength of mortar.

**Figure 10:**
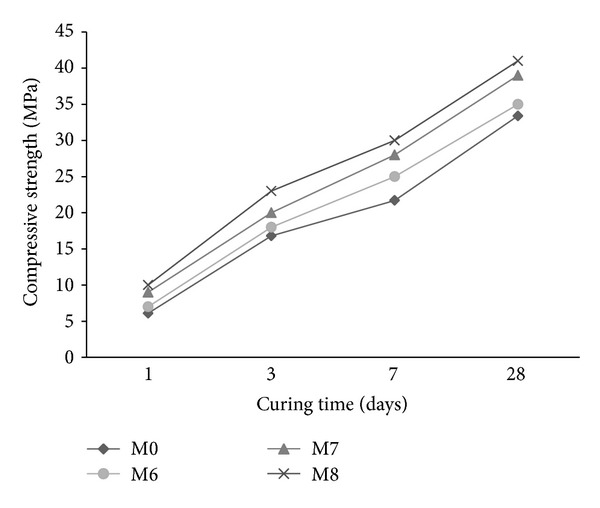
The effect of copolymer latexes prepared in presence of DBSS/POE on the compressive strength of mortar.

The compressive strength of mortar using synthesized copolymer latexes in presence of two cosurfactants DBSS/PVA and DBSS/POE is illustrated in Figures 9 and 10, respectively. The mortar containing latex St/MMA causes a reduction in compressive strength compared with the reference mix (M0). However, using latexes St/BuMA and St/GMA causes an increase in compressive strength (Figure 9). This may be attributed to the formation of a long side chain, which results in a polymer film that improves the strength [1].

The compressive strength values of mortar premixed with synthesized latexes in presence of POE are higher than those of the M0 as shown in Figure 10. Mortar with latex St/GMA exhibited the highest strength followed by mortar containing latexes St/BuMA and St/MMA, respectively. The increase in compressive strength is mainly attributed to the modification of bond between the cement pastes and sand due to the formation of a long side chain and the longer the chain, the higher the compressive strength [37–39].

From Figures 9 and 10 it is evident that mortars premixed with latexes in presence of PVA have a much lower compressive strength than that of POE. The decrease in the compressive strength of mortar with PVA seems to have been caused by the concomitant increase in air content arising from the surface activity of PVA [29]. On the other hand, PVA may have an adverse effect on the strength of the mortar because of the reduced latex film strength, the delayed cement hydration, and excess air entrainment. Similar results are reported by other authors [1, 29, 40].

### 3.6. Chemically Combined Water

**Figure 11:**
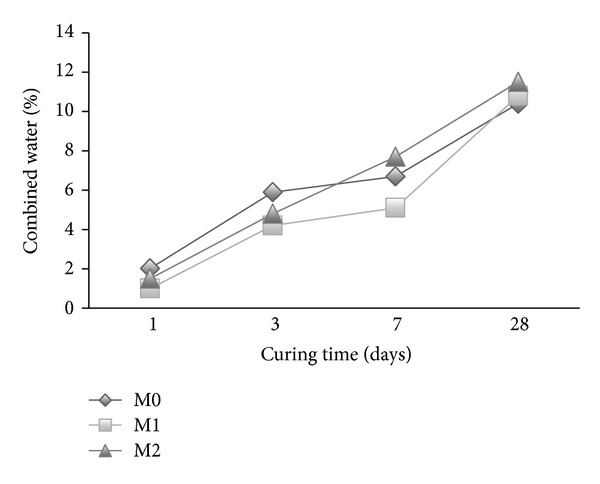
The effect of cosurfactants on the combined water content of mortar.

The chemically bound water for mortar with cosurfactants (M1 and M2) is shown in Figure 11. The combined water of the reference mortar M0 is higher than those containing cosurfactant during the first 7 days of hydration. Beyond 7 days, mortar with cosurfactant DBSS/POE exhibited higher combined water than the reference mortar M0 whereas mortar with DBSS/PVA has lower combined water than the reference. An increase in the amount of combined water is indicative of increase in the hydration product [41].

**Figure 12:**
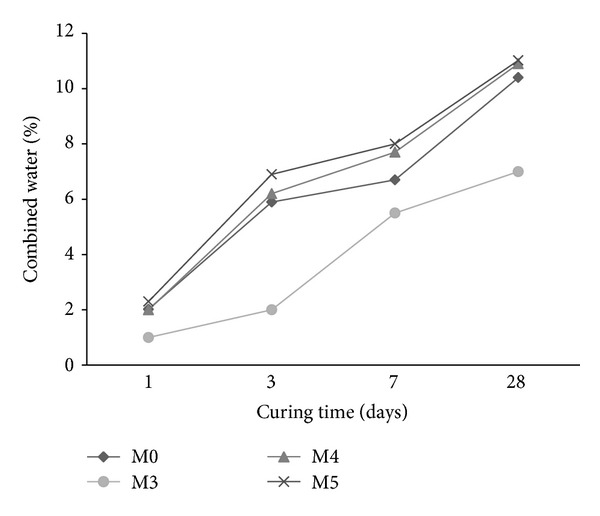
The effect of copolymer latexes prepared in presence of DBSS/PVA on the combined water content of mortar.

**Figure 13:**
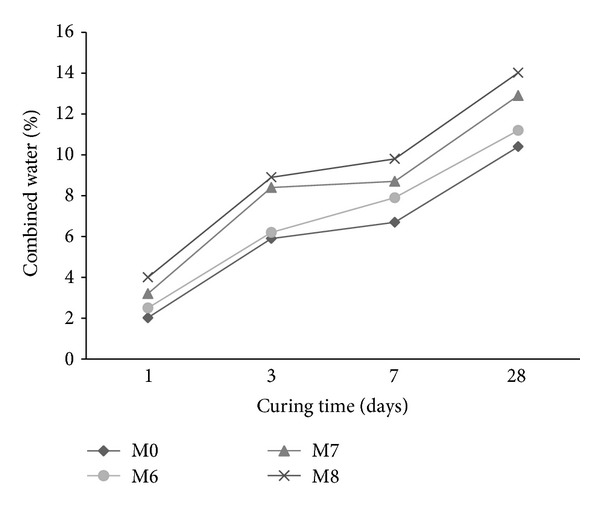
The effect of copolymer latexes prepared in presence of DBSS/POE on the combined water content of mortar.

The combined water of mortars premixed with copolymer latexes in presence of DBSS/PVA and DBSS/POE is shown in Figures 12 and 13, respectively. As it is clear, the combined water increases gradually as the curing time proceeds up to at least 28 days of hydration. This is mainly due to the continuous formation of the hydration products [42, 43]. The combined water content of the mortar premixed with St/MMA (M3) is lower than that of the other mixes (M0, M4, and M5) as shown in Figure 12. Furthermore, as the side chain of the copolymers increases in the presence of St/BuMA (M4) and St/GMA (M5), the combined water increases. The combined water of mortars with latexes in presence of POE is higher than that of the reference mortar M0 during the first 28 days of hydration. This is mainly attributed to the fact that the addition of copolymers did not form any films or membranes around the cement grains but only polymerizes or crystallizes inside the pore structure of the hardened cement pastes [42]. The combined water in case of polymer latexes in presence of POE had higher values than that of PVA during the first 28 days of hydration.

## 4. Conclusions

Based on the results obtained in this paper, the following conclusions can be drawn.

1. Flow of Portland cement mortar mixed with cosurfactants DBSS/PVA and DBSS/POE is higher than that of mortars containing latexes of these cosurfactants. Higher flow is caused by the creation of entrained air in the presence of cosurfactants.
2. The reduction in water consistency of mortars using copolymers in the presence of DBSS/POE is more than that of DBSS/PVA. This is associated with longer initial and final setting times and lower water absorption.
3. The compressive strength and chemically combined water for mortars with latexes containing DBSS/PVA are lower than those of DBSS/POE. This is attributed to the high content of voids in the presence of DBSS/PVA.
4. The longer side chains of the latexes gave more fluidity, a shorter setting time, lower water absorption, a higher strength and hydration.

## Figures and Tables

**Table 1:** The chemical structure of surfactants.

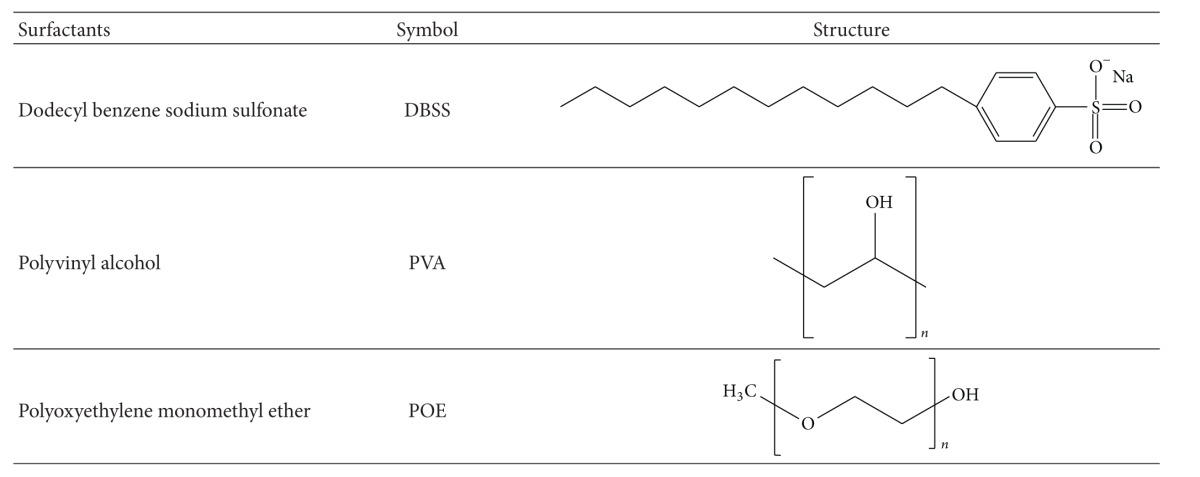

**Table 2:** The chemical composition of the raw materials, mass %.

Oxides materials	SiO_2_	Al_2_O_3_	Fe_2_O_3_	CaO	MgO	SO_3_	Na_2_O	K_2_O	L.O.I
PCC	21.48	6.03	4.22	64.29	0.68	0.39	0.21	0.11	1.32

G	0.58	0.14	0.11	30.08	0.13	45.36	0.07	0.09	22.16

**Table 3:** Admixtures used in mortar mixes.

Mix	Admixtures
M0	—

Cosurfactant	
M1	2% DBSS/1.5% PVA
M2	2% DBSS/1.5% POE

Copolymer latexes prepared in presence of 2% DBSS/1.5% PVA	
M3	St/MMA
M4	St/BuMA
M5	St/GMA

Copolymer latexes prepared in presence of 2% DBSS/1.5% POE	
M6	St/MMA
M7	St/BuMA
M8	St/GMA
